# Magnetic resonance imaging burn injury from metal eyelets on patient’s clothing

**DOI:** 10.1259/bjrcr.20230088

**Published:** 2023-10-24

**Authors:** Sarah Prescott, Bruce Jarvest, Harry Poole

**Affiliations:** 1 University Hospitals of North Midlands NHS Trust, Stoke-On-Trent, United Kingdom

## Abstract

This case report describes an incident which occurred following a cardiac MRI scan. The patient was permitted to wear their own jogging bottoms for the scan, which had two metal eyelets on them. The day after the MRI examination the patient called the MRI department to inform them that there was a lesion on their abdomen. The patient was assessed and this was diagnosed as a partial thickness burn. This case report describes the lessons learnt from this incident.

## Introduction

Magnetic Resonance Imaging (MRI) is usually considered to be a safe imaging modality, as it does not use ionising radiation. However, there are still risks associated with scanning, and it is essential that strict safety guidelines are followed to ensure that patients are kept safe.^1^ We report a rare case of a burn believed to be caused by a patient wearing trousers with metal eyelets during a cardiac MRI scan.

## Clinical presentation and investigations

**Figure 1.**
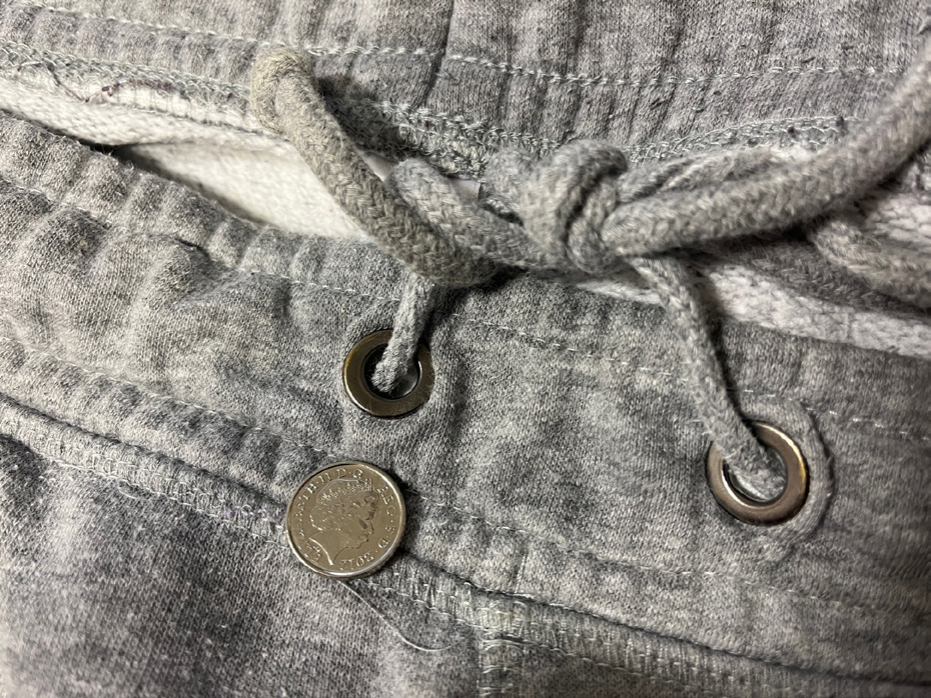
Patient’s jogging bottoms showing two metal eyelets, and a 5p coin to demonstrate size.

A 53-year-old patient underwent a cardiac MRI examination. Before the MRI scan, the department’s standard safety screening process was followed. This includes completion of the MRI safety screening form and, usually, changing the patient into a metal-free gown. This patient required a bariatric gown, but these were unavailable on the day of the scan. The patient was willing to remove their jogging bottoms; however, this was not done to preserve the patient’s dignity. The patient was, therefore, scanned in their own clothes, which included jogging bottoms with two metal eyelets (Figure 1). The radiographer noted that when the patient was setup on the table, the metal eyelets were not in contact with the patient’s skin, as there was a layer of material from the jogging bottoms and the patient’s underwear beneath. The radiographer genuinely felt the patient was adequately protected. However, it is believed that as the patient was moved into the scanner, some abdominal tissue had folded over the top of the jogging bottoms, and was therefore in contact with one of the metal eyelets.

The patient underwent a cardiac scan on a 1.5 Tesla MRI scanner (Siemens MAGNETOM Aera, Germany). They were positioned head first in a supine position on the 32-channel spine-phased array coil with an 18-channel body flex coil placed anteriorly. The total scan duration was 1 h and 3 min, excluding setup.

During the scan, the patient was scanned in normal specific absorption rate (SAR) mode throughout with a maximum whole body (WB) SAR of 1.9 W/kg. The radiographer was in regular contact with the patient throughout the scan. The patient did not report any pain or burning in the abdomen during or immediately after the scan.

The day after the examination the patient telephoned the department to say there was a lesion on their abdomen that was bleeding.

**Figure 2.**
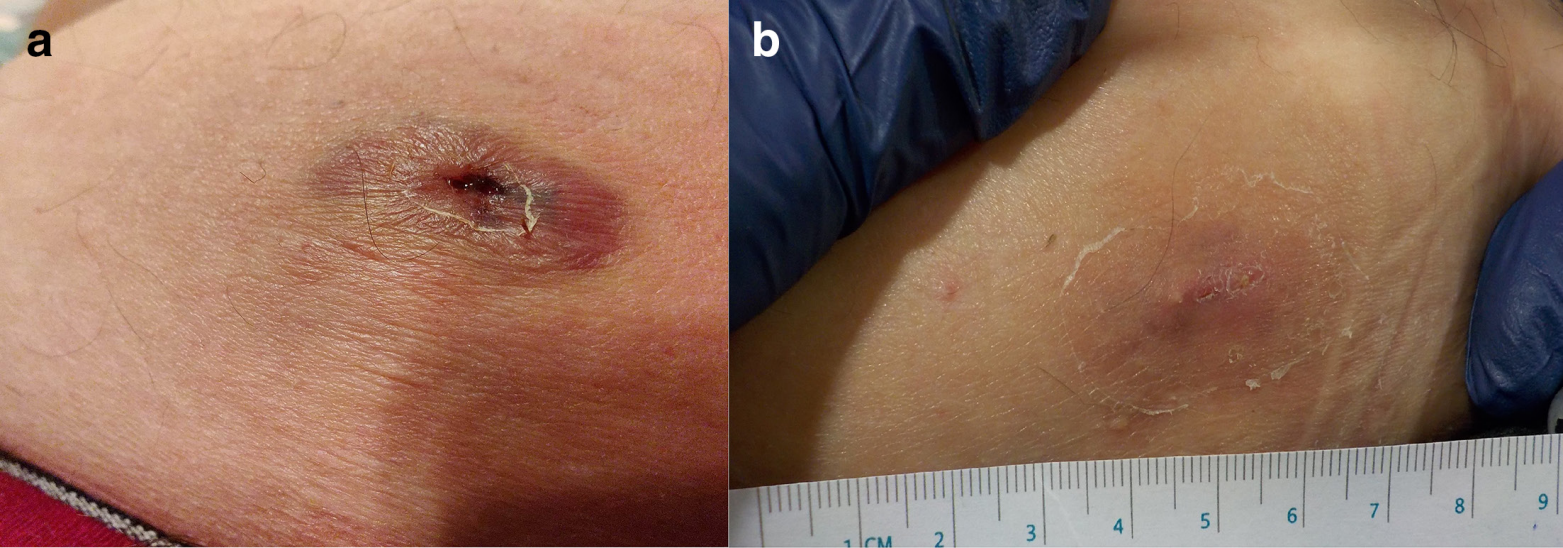
The appearance of the abdominal lesion at (**a**) day 6 and (**b**) day 13.

They were seen in the Central Treatment Suite (CTS) later that day where the patient was noted to have a 3 × 2 cm area of erythema with a central raw area and was diagnosed with a partial thickness burn. The patient was commenced on a course of oral antibiotics and was reviewed in the CTS clinic weekly until the wound was almost fully healed at 2 weeks. Figure 2 demonstrates the appearance of the lesion at day 6 and day 13. Four weeks after the MRI scan, the patient re-presented to the MRI department with new pain and swelling to the area. On examination, a small haematoma at the site of the previous burn was visible. The haematoma was evacuated, and a small sinus was visualised. The wound was dressed with inadine dressings, and the patient was seen on one further occasion in the CTS clinic 2 days later when they were discharged and continued to reapply dressings to the area at home.

## Discussion

Nearly, 4 million MRI scans are performed in England each year.^2^ MRI is usually considered to be a safe imaging modality, as it avoids the use of ionising radiation. The rate of reported incidents is extremely low, however, because of the strong magnetic field, rapidly switching magnetic gradients and radiofrequency pulses that are used, there are still some risks to the patient.^3^

In this case, it is believed that a thermal burn was caused by radiofrequency (RF)-induced tissue heating adjacent to a metal eyelet on the patient’s trousers. RF coupling of the two eyelets cannot be ruled out as a contributing factor in this case. Many previous reports highlight the risk of thermal burns.^4–7^ According to the Medicines and Healthcare products Regulatory Agency (MHRA), burns are the most commonly reported MRI-related adverse event in England.^1^ The MHRA guidelines^1^ state that burns can be avoided using careful patient positioning. They recommend that all patients are changed into metal-free clothing provided by the MR department before the scan. They also recommend that foam pads, 1–2 cm thick, should be used as insulation where there is a risk of heating. Clothing or blankets should not be used as a form of insulation. Neither of these recommendations were followed in this incident.

It is important that all MRI departments have MRI Local Rules to follow and that staff receive MRI safety training at induction, as well as at regular intervals during their career.

On the rare occasion that burns do occur, they are not necessarily immediately sensed by the patient,^1^ and therefore it is important that departments have policies in place which describe what to do if a patient presents with a potential burn after their MRI scan has taken place. In this incident, the patient was quickly followed-up by an appropriate specialist, which reduced the risk of further complications such as infection.

## Learning points

- MRI-related burns are a rare but serious complication that can arise if the correct precautions are not taken to prevent them.
- All patients must be changed into metal-free clothing before having an MRI scan, and appropriate patient screening must be in place.
- Where insulation is used to protect the patient, this must be in the form of foam pads at least 1 cm thick.
- All departments are encouraged to have a standard operating procedure (SOP) to follow in the rare event that an MRI-related burn takes place.
